# Two Lineages of *KLRA* with Contrasting Transcription Patterns Have Been Conserved at a Single Locus during Ruminant Speciation

**DOI:** 10.4049/jimmunol.1801363

**Published:** 2020-03-25

**Authors:** Mark S. Gibson, Alasdair J. Allan, Nicholas D. Sanderson, James Birch, Simon Gubbins, Shirley A. Ellis, John A. Hammond

**Affiliations:** The Pirbright Institute, Woking, Surrey GU24 0NF, United Kingdom

## Abstract

Cattle possess the most diverse repertoire of NK cell receptor genes among all mammals studied to date. Killer cell receptor genes encoded within the NK complex and killer cell Ig-like receptor genes encoded within the leukocyte receptor complex have both been expanded and diversified. Our previous studies identified two divergent and polymorphic *KLRA* alleles within the NK complex in the Holstein–Friesian breed of dairy cattle. By examining a much larger cohort and other ruminant species, we demonstrate the emergence and fixation of two *KLRA* allele lineages (KLRA*01 and -*02) at a single locus during ruminant speciation. Subsequent recombination events between these allele lineages have increased the frequency of KLRA*02 extracellular domains. KLRA*01 and KLRA*02 transcription levels contrasted in response to cytokine stimulation, whereas homozygous animals consistently transcribed higher levels of *KLRA*, regardless of the allele lineage. KLRA*02 mRNA levels were also generally higher than KLRA*01. Collectively, these data point toward alternative functional roles governed by *KLRA* genotype and allele lineage. On a background of high genetic diversity of NK cell receptor genes, this *KLRA* allele fixation points to fundamental and potentially differential function roles.

## Key Points

- Two divergent allele lineages of *KLRA* have become fixed in the cattle population.
- The two cattle *KLRA* allele lineages likely have contrasting functions.

## Introduction

Natural killer cells are a population of large granular lymphocytes with essential functions in immunity, cancer, and reproduction (1). NK cells are early responders to infection, particularly against viruses, and are capable of recognizing and killing infected and transformed host cells as well as initiating subsequent immune responses through the secretion of cytokines. This cytokine release helps to drive adaptive immune responses led by B and T cells, which can be augmented by direct interactions between NK cells and other APCs or restricted by direct interactions with T cells (2–4). NK cells also play a fundamental role in human reproduction during the formation of the placenta through cooperation with the extravillous trophoblast (1).

In humans and rodents, NK cells are a highly heterogeneous population with different functions, specificities, and activation thresholds (5, 6). This functional diversity is largely driven by the variegated expression of polygenic and polymorphic germline encoded receptors, a majority of which recognize polymorphic determinants of MHC class I. These receptors can be either activating or inhibitory, and the balance of signals received when these receptors are cross-linked with their ligand determines the functional potential and activation status of an individual NK cell. This NK cell receptor and MHC class I diversity is now known to influence differential outcomes to several viral infections and is associated with a number of autoimmune diseases (7–11).

The diversification of NK cell receptor gene families has occurred independently several times during mammalian evolution. There are two classes of NK cell receptors in mammals that are known to have diversified: the killer cell Ig-like receptor (KIR) family located within the leukocyte receptor complex (LRC), and the structurally unrelated killer cell lectin-like receptors (KLR) located within the NK complex (NKC). A diverse *KIR* gene repertoire is a feature of prosimian primates, including humans, apes, and Old and New World monkeys. In contrast, lemurs have expanded the *KLRC/D* gene families (12), whereas rodents and horses have expanded the *KLRA* family (13, 14). Cattle were thought to be unique in having expanded LRC and NKC gene families, the *KIR* (15), and the *KLRC/D* (16), respectively. However, recent studies have confirmed other ruminant species have also expanded both NK cell receptor gene complexes (17, 18).

The ligands for a majority of the primate KIR and rodent KLRA are the highly diverse classical MHC class I (19). These receptor–ligand systems are considered equivalent to each other in that they generate NK cell functional diversity in a rare example of convergent evolution. Although the ligands for these receptors in other species have yet to be confirmed; considering the genomic synteny, homologous gene expansion mechanisms, and gene sequence conservation, it seems likely that these other diversification events are also examples of convergent evolution being driven by selection to produce NK cell functional heterogeneity. We have previously shown that the *KIR* and *KLRC/D* are differentially transcribed between cattle NK cells and that this mRNA expression is influenced by the MHC genotype (20). This offers strong evidence that this highly diverse receptor gene repertoire in cattle also creates functional diversity.

Against this background of *NKC* and *LRC* gene expansion, cattle *KLRA* appears to have remained functional and monogenic, but there are at least two distinct gene lineages (21, 22). Two novel *KLRA* transcripts were identified that significantly differed between the extracellular lectin domains from the genome reference sequence, creating the potential for differential ligand binding and functional diversity (22). The KLRA receptors are homodimeric C-type lectin type II transmembrane glycoproteins. In mice, this includes at least 23 members (Ly-49A-W), with different mouse strains having a specific complement of genes (23). Not all these genes are functional; of those that are, most are inhibitory, several are polymorphic, and they generally demonstrate broad recognition of MHC class I or recognize defined class I subsets, as is the case with KIR in primates (24–28).

To further understand if cattle *KLRA* diversity was indicative of limited gene expansion or a highly polymorphic gene, we confirmed the structure and polymorphism of the *KLRA* locus in cattle and closely related species and examined *KLRA* lineage frequency within two cohorts of Holstein–Friesian bulls used as pedigree sires for dairy farmers. To assess the potential functional impact of *KLRA* diversity, we analyzed the relative NK cell mRNA expression of both allele lineages within and between individuals with different *KLRA* genotypes under cytokine stimuli. Cattle are an essential food-producing species subject to intense selective breeding in a global effort to increase food production while protecting the climate. Understanding the immunogenetic diversity of livestock species is a fundamental part of this effort by underpinning breeding strategies that reduce disease burden and increasing vaccine efficacy.

## Materials and Methods

### Ethics statement

Peripheral blood samples from Holstein–Friesian cattle (*Bos taurus*) were collected in accordance with the U.K. Animal (Scientific Procedures) Act, 1986, and approved by either The Pirbright Institute Ethics Committee, or The Roslin Institute’s Animal Welfare and Ethics Committee. The Chillingham cattle samples were taken from animals culled for welfare reasons. Blood sampling of Kuchinoshima-Ushi cattle was carried out in accordance with the Regulations for Animal Experiments in Nagoya University and the Guidelines for the Care and Use of Laboratory Animals by the Tokyo University of Agriculture. For all the other species, blood samples taken during necessary veterinary interventions were kindly supplied by The Zoological Society of London.

### Transfection, flow cytometry, and quantification of KLRA surface expression

The full coding sequence of KLRA*01 and KLRA*02 were amplified using cDNA from animals previously identified as being homozygous using primers sense Ly-49HindIII (5′-CTGCTGAAGCTTACCATGAGTGATCAAGAAGTG-3′) and antisense XhoI (5′-GTAGTACTCGAGGTGTTTATTGAAGCAATC-3′). PCR products were cloned into TOP10 *Escherichia coli* using the TOPO cloning system (Thermo Fisher Scientific, Renfrew, U.K.) and Sanger sequenced to identify clones with no errors. Inserts were digested from identified clones using HindIII and XhoI and ligated into the pcDNA6/V5-His vector (Thermo Fisher Scientific) and electroporated into P815 cells using standard protocols. Expanding clones were grown under blasticidin selection and picked for further expansion after 3–5 d. Cultures derived from the clones were screened by flow cytometry using an Ab against the V5 tag (V8012; Merck, Dorset, U.K.). Expression was confirmed in positive clones by Western blot using the V5 Ab to detect a protein of the correct size.

### KLRA PCR amplification from genomic DNA

The genomic sequences of *KLRA* from a range of ruminant species (cattle, African buffalo, Bactrian camel, European bison, gemsbok, giraffe, greater kudu, lowland anoa, moose, red deer, water buffalo, and yak) were aligned. Primers (Sigma-Aldrich, Gillingham, U.K.) were designed against unique, allele-specific regions of KLRA*01 and KLRA*02, and for KLRA*01/02, a region shared with KLRA*02. Primers were selected in regions conserved between all relevant species: sense BotaKLRA1_SSP1 5′-GCCAAAGTCACAATTCGTG-3′, sense BotaKLRA2_SSP2 5′-GCCAAAGTCACGATTCATC-3′, and antisense KLRA 5′-CTCCAAGAAGCAACTGCCACTCC-3′.

These primers were used, where appropriate, to genotype all animals in this study for the presence/absence of KLRA*01, KLRA*02, and KLRA*01/02. PCR amplification of genomic DNA was performed with GoTaq (Promega) for 40 cycles using a minimum of 20 ng DNA. The complete amplified KLRA*02 genomic DNA sequence was submitted to GenBank (accession number MH991705; https://www.ncbi.nlm.nih.gov/nuccore/MH991705).

### Genomic enrichment of the cattle KLRA locus, mapping, and variant calling

The variant data used to analyze single nucleotide polymorphisms (SNPs) in this manuscript were generated from enriched genomic DNA samples produced in (29). Briefly, fragmented genomic DNA was used to generate indexed Illumina TruSeq sample libraries, which were then enriched using a custom set of SeqCap EZ oligonucleotide probes (Roche Sequencing). Four libraries were pooled and sequenced on an Illumina MiSeq (2 × 250-bp paired-end reads). In addition to variant calling using SAMtools, the SAMtools phase function was used on the BAM file for each mapping (30), which is a relatively conservative estimate of different haplotypes, as it requires evidence for phasing within individual reads.

### Isolation of cattle NK cells from peripheral blood

NK cells were positively selected from Holstein–Friesian PBMCs by labeling with anti-CD335 mAb recognizing bovine NCR1 (AbD Serotec, Kidlington, U.K.) and rat anti-mouse IgG1 MicroBeads (Miltenyi-Biotec, Bergisch Gladbach, Germany) to purities >85%. After washing in RPMI 1640, cells were either cultured with cytokines or frozen for RNA extraction.

### Cytokine stimulation assays

NK cells were isolated as described above and cultured in RPMI 1640 supplemented with 10% heat-inactivated FCS, 10 mM HEPES, and 50 μM 2-ME (Sigma-Aldrich with 10 U/ml penicillin/streptomycin) (Microbiological Services, Pirbright, U.K.). The cytokine regimes were added to the media, either recombinant bovine IL-2 (0.5 μg/ml), recombinant human IL-12 and 18, or IL-15 (0.1 μg/ml). Cells were cultured for a maximum of 7 d in 24-well plates (Thermo Fisher Scientific) at 37°C in a 5% CO_2_ incubator with serial splitting occurring when cell confluence was reached.

### Generation of NK cell cytokine-stimulated clones

Isolated NK cells were stimulated with rbIL-2 or rhIL-15 (same conditions as above) for 24 h prior to limiting dilution assays using 5 × 10^4^ allogeneic irradiated PBMC feeder cells per well. We did not measure if any cytokines were being produced by the feeder cells, but NK cells with feeder cells without cytokine in the media did not proliferate. Cytokine-stimulated cultures were incubated for 14 d. Growing cultures were expanded on 1 × 10^6^ fresh feeder cells, and NK cell origin was confirmed by labeling with an anti–CD335-PE (AbD Serotec) Ab. TRT1 (IgG1) (courtesy of Microbiological Services) was used as an isotype control mAb. Flow cytometry was performed using the LSRFortessa II (Becton Dickinson, Oxford, U.K.) and analyzed using FCS Express v3 (De Novo Software, Los Angeles, CA). A minimum of 20,000 events were collected, and positive staining was determined by matching isotype control samples.

### RNA isolation, cDNA synthesis, and quantitative PCR analysis of cattle KLRA transcription

Total RNA was isolated from 3 × 10^6^ cytokine-stimulated NK cells or NK cell clones using TRIzol reagent (Life Technologies) according to manufacturers’ instructions. cDNA was synthesized using SuperScript II with oligo (dT_12–18_) primers (both Life Technologies). Quantitative PCR was used to assess the mRNA levels of cattle *KLRA*. Assays were performed using Luminaris HiGreen low ROX qPCR Master Mix (Thermo Fisher Scientific) with 250 ng cDNA per reaction. Thermocycling conditions were 2 min at 50°C, 10 min at 95°C before 40 cycles of 15 s at 95°C, and 1 min at 60°C. An additional heating step was run from 60 to 95°C to obtain melting curves. The primers for ATP5B (sense: 5′-CCTTCTGCTGTGGGTTATCA-3′; antisense: 5′-CAGGATCCGTCAAGTCATCA-3′), EIF2B2 (sense: 5′-GAGCATATCCACTCCAACGA-3′; antisense: 5′-CACTCTGCCACAATGACATG-3′), and SDHA (sense: 5′-GCTCTCCTACGTTGACATCA-3′; antisense: 5′-AAGCCTCAGTCTCCTCAGTA-3′) were designed and validated in-house (obtained from Sigma-Aldrich) using the geNorm^PLUS^ application in the qbase^PLUS^ software version 2.5.1 (2, 3). These primers were applied at a concentration of 300 nM. Primers for KLRA*01 and KLRA*02 were custom designed using Locked Nucleic Acid bases (Exiqon A/S, Vedbaek, Denmark) and used at a concentration of 900 nM. All primer efficiencies lie within the range of 90–110%.

### Statistical analysis

Statistical analyses were performed in R v. 2.15.2 (http://www.r-project.org). Expression levels for each animal were first normalized using three endogenous reference genes (ATP5B, EIF2B2, and SDHA) (20) and then against the mean normalized expression level across either all NCR1-positive samples (for the cytokine stimulation data) or all samples (for the ex vivo cultures). Differences in KLRA expression levels among ex vivo cell populations were analyzed using a linear mixed model for each gene of interest, with log_2_ normalized expression levels as the response variable, cell population (PBMC, NCR1^+^, or NCR1^−^), and genotype (homozygous or heterozygous) as fixed effects and animal as a random effect. The effect of cytokine stimulation on KLRA expression levels was analyzed using a linear mixed model for each gene of interest, with log_2_ normalized expression levels as the response variable, cytokine (unstimulated NCR1^+^, IL-2, IL-12/18, or IL-15) and genotype (homozygous or heterozygous) as fixed effects and animal as a random effect. For both analyses, significant (i.e., *p* < 0.05) factor levels were compared using Tukey honest significant differences.

## Results

### The two divergent allele lineages of cattle KLRA are the products of a single locus

Cattle contain a unique and expanded NK cell receptor gene repertoire that creates diversity in the NK cell population. We previously reported that although there is only one *KLRA* gene in the cattle genome assembly (derived from a Hereford), Holstein–Friesian cattle possess two very distinct *KLRA* allele lineages: Ly-49*01 and Ly-49*02/3 (22). To examine the relationship between these divergent alleles, we used an overlapping exon to exon genomic DNA PCR strategy to amplify the entire *KLRA* gene–encoding Ly-49*02 and compared this to the known *KLRA* (Ly-49*01) within the genome assembly. Both genes are almost identical in length at ∼15 kb, but 235 SNPs and several small indels create a pairwise sequence identity of 97%. PCR amplification of the full coding cDNA from 15 unrelated Holstein–Friesians never identified more than two alleles from one individual. This strongly implies that the sequences previously described as Ly-49*01 and Ly-49*02/03 are the divergent products of a single *KLRA* locus, subsequently referred to as the KLRA*01 and KLRA*02 allele lineages.

### The coding sequence of both KLRA lineages are conserved despite genomic recombination

Full-length cDNA analysis from the 15 animals above only identified two new KLRA*01 alleles that both differed from KLRA*01 by a single synonymous substitution. This lack of minor variants was surprising considering the extent of polymorphism between KLRA*01 and -*02. To study the entire locus in more detail, we used probe-based genome enrichment to pull down the complete *KLRA* gene and surrounding region of the cattle genome from 17 Holstein–Friesian cattle, three feral *B. taurus* (two Chillingham and one Kuchinoshima-Ushi), and three animals from the closely related *B. indicus* subspecies (two Nelore and one Sahiwal) that diverged from *B. taurus* 1.85 ± 0.15 million y ago (mya) (31). Included in the Holstein–Friesian cohort were 11 animals that had already been analyzed by PCR and were known to be KLRA*01 or KLRA*02 homozygous or heterozygous. The enriched genomic fragments were sequenced and mapped to the extended *KLRA* locus in the cattle genome. The overall high identity between KLRA*01 and KLRA*02 allowed the reads corresponding to either allele group to map with high confidence.

**FIGURE 1.**
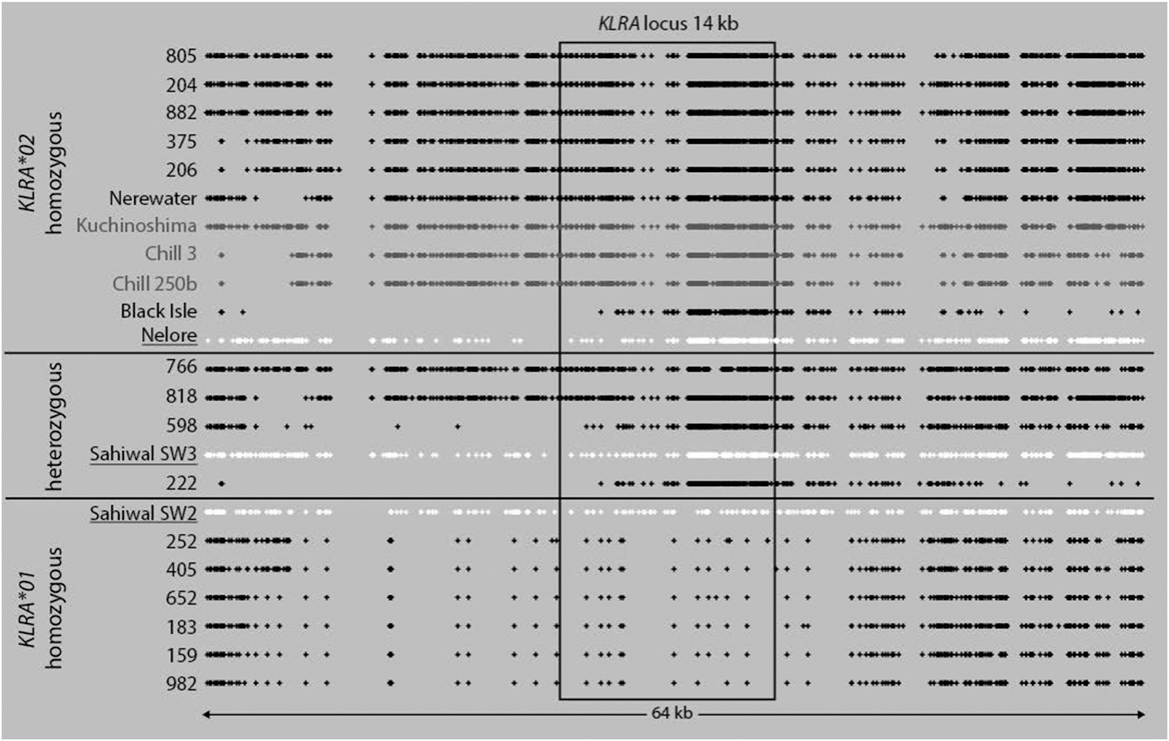
SNP diversity over the extended *KLRA* locus. Enriched genomic data from *B. taurus* (feral species names and SNPs in gray) and three *B. indicus* (names underlined and SNPs in white) were mapped to the *KLRA* locus in the UMD3.1 cattle genome. SNPs are indicated with a dot, and *KLRA* genotype is given according to the previously established or predicted cDNA sequencing.

Enriched genomic sequence data from all the animals, including the feral *B. taurus* and *B. indicus* animals, mapped relatively uniformly over the entire *KLRA* locus and ∼100 kb either side. Within the 14 kb *KLRA* gene, there were four intronic regions not represented by any sequence data, which in total accounted for <1.1 kb of the gene. These regions were all of low complexity or very repetitive and were not conducive to initial probe design. Of the 100 kb either side of the *KLRA* locus analyzed, a 64 kb region encompassing the *KLRA* locus contained a highly variable SNP pattern between animals (Fig. 1).

The allele content of each animal could clearly be determined based on the known exonic SNPs that distinguish both *KLRA* lineages. Based on mapped exon sequences alone, seven animals were homozygous for KLRA*01, thirteen were homozygous for KLRA*02, and five appeared to be heterozygous (Fig. 1). Data from Holstein–Friesian animals with a known *KLRA* allele content were entirely consistent with the predicted genotype, based on our previous cDNA analysis. However, the SNP patterns at the 5′ end of the gene indicated recombination had likely occurred. In an attempt to more accurately decipher the allele content from heterozygous animals and locate potential regions of recombination, the sequence data from each animal were phased to distinguish both alleles. This method uses SNPs between two genome copies to allocate an individual read to one allele or another. The number of SNPs over this region is relatively high in a whole-genome context. Accordingly phasing successfully separated KLRA*01 and KLRA*02 in animals that were previously known to be heterozygous except for animal 766. This animal was a twin and appeared to contain more than two *KLRA* alleles as well as other regions of the NKC. A vascular connection between the placentas of cattle twins and the sharing of genetic material is a well-known phenomenon, and therefore, this animal was excluded from further analysis. As the sequences were not entirely contiguous because of the gaps in the pulldown probe coverage, assembled regions were manually labeled as KLRA*01 or KLRA*02 based on identity to the known genomic sequences. The sequences of these contigs were then compared with the same assembled reads for the homozygous animals to provide confidence that they were from the correct *KLRA* allele lineage. It is, however, possible that a recombination point within one of these low complexity regions has been overlooked.

**FIGURE 2.**
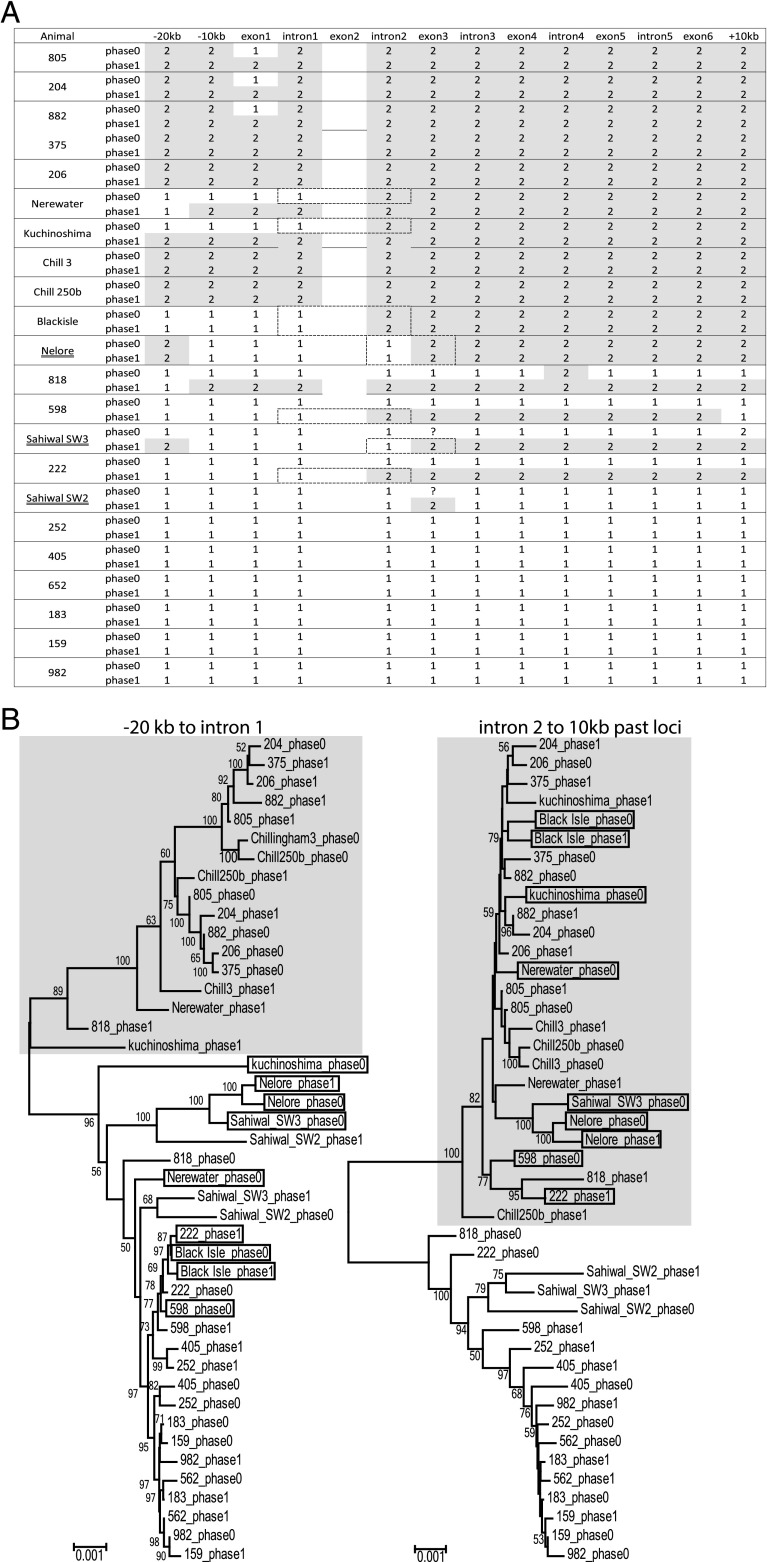
Phased *KLRA* alleles from each animal indicate a common recombination point. (**A**) Enriched genomic data were phased and broken down by intron and exon for phylogenetic analysis. Assignment to KLRA*01 or KLRA*02 is indicated by a 1 or 2 (shaded), respectively. *B. indicus* animal names are underlined. Predicted recombination points are boxed with a broken line. (**B**) Neighbor joining phylogenetic trees drawn using Molecular Evolutionary Genetics Analysis software (39) with 1000 bootstraps. Branch support is shown, and the clear grouping between the KLRA*01 and KLRA*02 can been seen. The 5′ and 3′ trees were constructed separately, excluding exon 2 as this was the predicted recombination point. The KLRA*02 group is shaded, and the recombinant alleles are boxed.

Domain-by-domain phylogenetic analysis with the phased allele sequences clearly highlighted two regions where recombination between KLRA*01 and KLRA*02 had occurred (Fig. 2). Analysis of SNP patterns revealed that, in the *B. taurus* animals, there was a break point within a 198-bp region of intron 1–100 bp upstream of exon 2 (positions 4518–4690 in accession number MH991705; https://www.ncbi.nlm.nih.gov/nuccore/MH991705) characterized by adenine cytosine repeats, and in *B. indicus*, the break point was within a 174-bp region in the middle of intron 2 (positions 1571315-1571118 in accession number KX592814; https://www.ncbi.nlm.nih.gov/nuccore/KX592814) characterized by 66% adenine thymidine content. The same recombinant alleles were seen six times in the taurine and three times in the indicine cohort, indicating that these alleles are common in both groups. However, as the break point is in a different place between these species, these were likely independent gene conversion events. As some intronic sequence was missing, the phasing was not contiguous across the whole allele, but phylogenetic analysis of the data were entirely consistent with the alleles present at the 5′ end of the gene. It is, therefore, interesting that the consequence of this recombination in both species has been to increase the frequency of the KLRA*02 extracellular domains, as all these recombinant alleles essentially encode an allele of KLRA*02.

To examine the genomic context of this *KLRA* allele conservation on a background of recombination, we analyzed the phased sequences associated with each allele lineage up to 100 kb either side of the *KLRA* locus (Supplemental Fig. 1). Our analysis and the current genome builds do not locate any other genes within this region. Phylogenetic analysis of the 100-kb region centromeric of the *KLRA* gene divides the KLRA*01 and -*02 alleles into two distinct clades, with the recombinant alleles falling within KLRA*01, as expected from the domain-by-domain analysis. Within the KLRA*01 clade, there are three distinct branches, one containing all the *B. indicus* animals and another more divergent, but distinct, clade containing six Holstein–Friesian and one Kuchinoshima allele (Supplemental Fig. 1). The clear division between the KLRA*01 and -*02 alleles breaks down after the coding region, with less than half the allele sequences from each lineage cladding together. It is clear that the recombination in this region of the genome has been more frequent and complex.

The high resolution of the enrichment data made it possible to characterize allelic variation. In the *B. taurus* animals, at least seven KLRA*01 and four KLRA*02 minor alleles were present, with an average of 199 SNPs between each lineage, which is entirely consistent with our previous whole-gene amplification. All the KLRA*01 and KLRA*02 allele lineages sequenced in this study can be distinguished from each other between 213 and 223 SNPs over the entire 14 kb of the locus, 18 of which are in the exons, as previously reported (22), and remain completely invariable in this study. Within the Holstein–Friesian dataset, each allele lineage is remarkably conserved, with 16 and 20 variable nucleotide positions between KLRA*01 and KLRA*02 alleles respectively, none of which are located in the exons. Although there has clearly been diversification of at least two *KLRA* allele lineages, because they shared a common ancestor, they appear to have become fixed in the Holstein–Friesian population.

### KLRA allele lineages predate cattle speciation and have been conserved for millions of years

**FIGURE 3.**
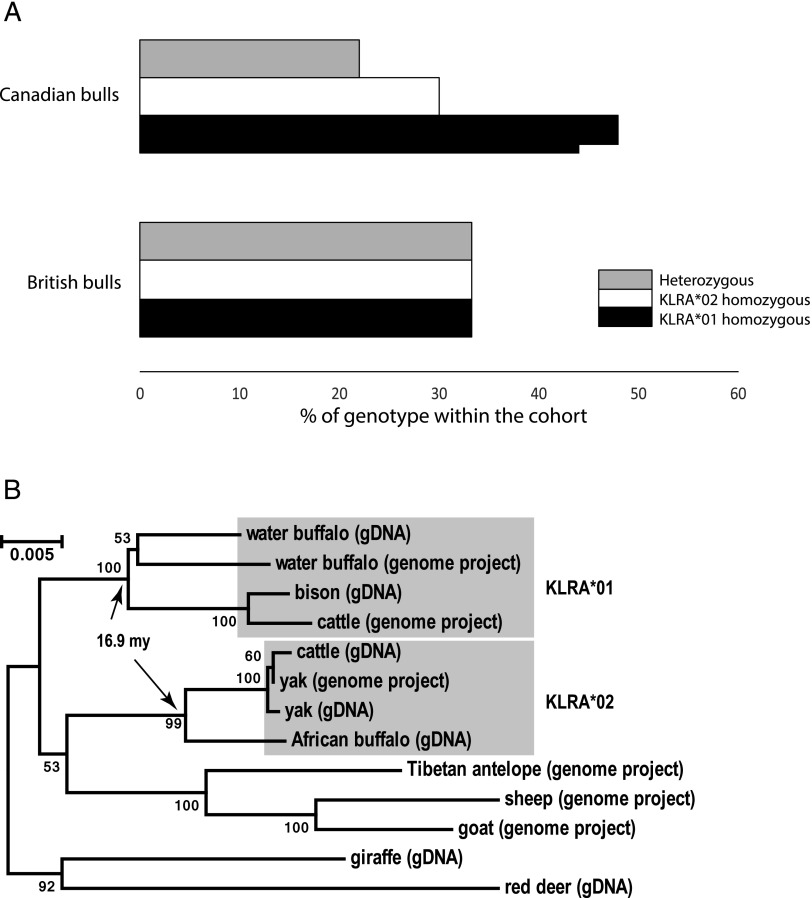
KLRA allele lineage frequency and diversification. (**A**) The *KLRA* genotype of a group of British and Canadian bulls was determined by PCR. KLRA*01- and KLRA*02-specific primers were used to establish the lineage frequency within a cohort of 73 animals. The different genotypes in each population of bulls are represented as a percentage of the total number of animals assessed within that population. Black and white bars represent KLRA*01 and KLRA*02 homozygous animals, respectively. Gray bars represent heterozygous animals. (**B**) *KLRA* allele lineage diversification was investigated in a group of 10 ruminant species. Intron 4 was amplified by PCR from genomic DNA samples in seven of the species, as indicated, and PCR products were sequenced. The *KLRA* sequence was acquired from six of the species examined in which full genome sequences were available, as indicated. Intron 4 sequences from all 10 ruminant species were aligned to produce neighbor-joining phylogenetic trees using Molecular Evolutionary Genetics Analysis software with 1000 bootstraps. Branch support is indicated at each node.

Modern Holstein–Friesian dairy cattle genetics are dominated by relatively few elite bulls, and their offspring that have been selected largely for production traits. To establish the frequency of the *KLRA* lineages in representative bulls, we PCR typed DNA from a group of elite British and Canadian bulls. Genotyping by PCR using KLRA*01 and -*02 lineage–specific primers revealed a higher frequency of homozygous genotypes than expected from a random distribution (Fig. 3A). This was particularly striking in the Canadian bull cohort, in which 50% of all animals were homozygous for KLRA*01, with only 22% heterozygous. This suggests that there has been positive selection for *KLRA* homozygous animals, although it is not yet clear if this has been recent artificial selection of breeding bulls. This supports our previous data, as there does appear to be an advantage to maintaining both allele lineages in the population.

To better resolve the evolutionary history of *KLRA* allele diversification, we amplified intron 4 of the *KLRA* gene from representative ruminant species and took advantage of sequencing data from ruminant genome projects. Intron 4 is 2.2 kb and the most variable between lineages. Phylogenetic analysis shows that well before the speciation of cattle, these lineages were clearly distinguishable in species that shared a common ancestor ∼17 mya and suggests that the KLRA*02 lineage may be more like the ancestral sequence (Fig. 3B). Although cattle are the only single species in which we have found evidence of both lineages, we cannot be certain this is unique because of the limited numbers of other species used in this study. However, it is clear that these lineages have persisted for at least 17 mya, and we found no evidence of gene duplication.

### KLRA is predominantly transcribed by NCR1^+^ lymphocytes and responds to cytokine stimulation

Lineage conservation suggests an important functional role for KLRA on bovine lymphocytes. To confirm that both alleles have the potential to be expressed on the cell surface, P815 cells were transiently transfected with an expression construct containing KLRA*01 or KLRA*02 that incorporated the V5 epitope on the extracellular C-terminal domain. Flow cytometry confirmed that both allele lineages are trafficked to the cell surface by the intracellular machinery and are likely functional (Supplemental Fig. 2).

**FIGURE 4.**
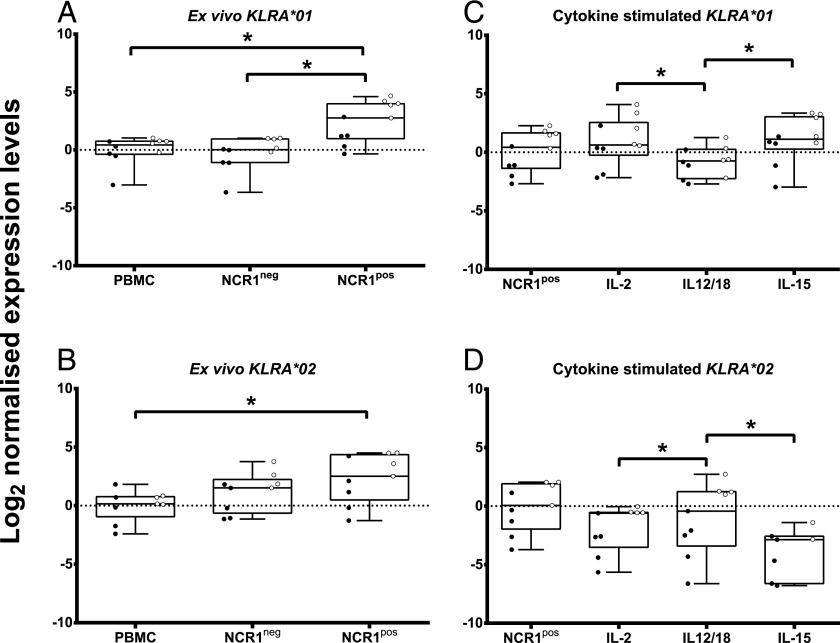
*KLRA* transcription is predominant in NCR1^+^ cells over other lymphocytes, and transcription within NCR1^+^ cells is influenced by different cytokines. KLRA*01 (top) and KLRA*02 (bottom) transcription was assessed in ex vivo (**A** and **B**) PBMCs, NCR1^–^ and NCR1^+^ cells from 14 animals. In addition, ex vivo NCR1^+^ cells were compared with NCR1^+^ cells that had been stimulated for 7 d in vitro with IL-2, IL-12, and IL-18 or IL-15 (**C** and **D**). Statistical significance is indicated between samples. Open circles represent homozygous animals, and filled circles represent heterozygous animals. *p < 0.05.

To explore expression further, mRNA from ex vivo PBMCs isolated from 14 Holstein–Friesian cattle of known *KLRA* genotype (12 were included in the genome enrichment experiment) was assessed for *KLRA* allele transcription using quantitative PCR. As KLRA receptors are typically found on NK cells in mice (32), ex vivo NCR1^+^ cells [the key differentiator of NK cells and a subset of NKT-like cells in cattle (33)] and NCR1^+^-depleted lymphocytes were also assessed. These analyses demonstrated that both *KLRA* lineages were transcribed, with mRNA significantly (*p* < 0.001) more likely to be transcribed by NK cells (Fig. 4A, 4B, Supplemental Table I). To interrogate whether *KLRA* lineage expression was likely to vary during an immune response, NCR1^+^ cells were stimulated using three different cytokine regimes. IL-2 and IL-15 primarily promote cell division and homeostasis, whereas IL-12 and IL-18 in synergy induce NCR1^+^ cells to secrete IFN-γ and produce cytotoxic granules. Surprisingly, KLRA*01 and KLRA*02 transcription levels contrasted (Fig. 4C, 4D, Supplemental Table II). Between stimulated samples, KLRA*01 transcription increased after incubation with IL-2 or IL-15 in comparison with IL-12/18. In contrast, KLRA*02 transcription was reduced by the same cytokines, with IL-12/18 having no significant effect. The two *KLRA* lineages show the opposite transcriptional response to IL-2 and IL-15 stimulation on NCR1^+^ cells.

### KLRA lineage copy number correlates with transcription

**FIGURE 5.**
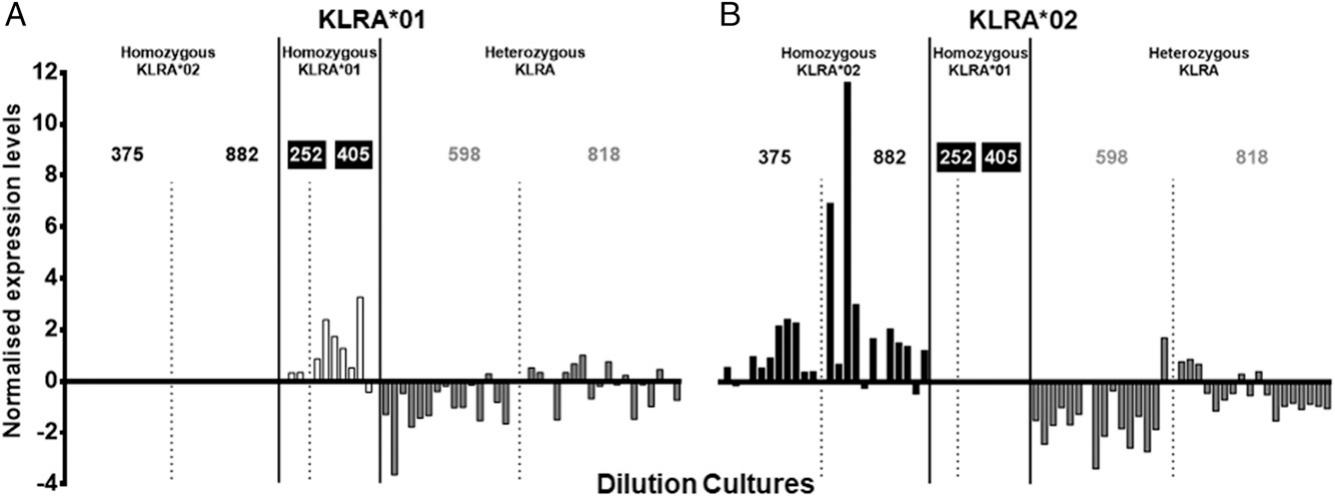
*KLRA* allele transcription exhibits an allelic dosage effect, which is dependent upon *KLRA* genotype. Sixty-seven single-cell dilution cultures were generated from six animals: two animals were homozygous for KLRA*01 (252 and 405; white bars), two homozygous for KLRA*02 (375 and 882; black bars), and two were heterozygous for *KLRA* alleles (598 and 818; gray bars). Dilution cultures were assessed for transcription of KLRA*01 (**A**) and KLRA*02 (**B**). Each bar represents a single dilution culture, and samples from individual animals are separated by a dashed line, with *KLRA* haplotypes separated by a solid line.

Both KLRA*01 and KLRA*02 homozygous animals showed significantly higher transcription than heterozygous animals, immediately ex vivo and after cytokine stimulation, with homozygous animals transcribing 3.5- to 8-fold more than homozygous animals (Fig. 4A, 4B). To examine this and the variable expression between each *KLRA* lineage in more detail, mRNA from a previously generated set of 67 single-cell NCR1^+^ dilution cultures (20) was examined using quantitative PCR. These cultures were derived from six animals that were also included in the genome enrichment experiment. Both *KLRA* alleles were differentially expressed by individual dilution cultures within and between individuals with 7-fold and 16-fold differences in expression levels for KLRA*01 and KLRA*02, respectively (Fig. 5). This limited sample set also indicated a gene dosage effect may be apparent, with dilution cultures from homozygous animals transcribing more *KLRA* than those from heterozygous individuals and higher transcription of KLRA*02. This all points to a functional difference between cells from homozygous and heterozygous individuals that may be linked to the overrepresentation of homozygous individuals in the Holstein–Friesian breeding bulls.

## Discussion

This study examines *KLRA* complexity in a large and diverse cohort of cattle to develop a greater understanding of its potential importance in cattle immune responses. We previously described two distinct *KLRA* allele lineages at the cDNA level (22) and, in this study, found lineage divergence extends across the entire length of both nucleotide sequences at the gene level. Consistent with previous studies (21), we found no evidence of gene duplication in cattle, confirming these allele lineages are transcribed from a single *KLRA* locus. Lineage-segregating patterns of polymorphism were identified, including 18 stable coding region SNPs identical to those found previously (22). This provided strong evidence these lineages have become fixed during cattle evolution, suggesting they have emerged as beneficial and retain important functional roles. The yak and water buffalo genome sequences contain 17 and 9 of these 18 coding region SNPs, respectively, confirming that this allele diversification predates cattle speciation and has become fixed.

Phasing the enrichment data identified two recombination hot spots toward the 5′ end of the gene. It is interesting to observe that although the exact locations of these two breakpoints differ, they remain relatively close to one another within the gene. The net outcome of these independent conversion events is an enrichment of KLRA*02 extracellular domains, presumably ligand binding, in both recombinant allele groups. Fifteen out of the twenty-two animals used in this study have at least one KLRA*02 lineage allele despite random sampling. Although the KLRA*02 lineage was also the most common in our previous study (22), analysis was restricted to Holstein–Friesian cattle. From selecting a more genetically diverse group of cattle in this study, we found KLRA*02 lineage alleles in every feral *B. taurus* and two of the three *B. indicus* animals examined, several of which were recombinants.

In the two groups of breeding bulls studied, we found strong evidence of allelic bias with a greater prevalence of homozygous animals of both *KLRA* allele lineages. A similar trend was apparent in the animals we randomly selected for genome enrichment. There is presumably a fitness benefit to both allele lineages persisting in the population, which may explain why the frequency of both homozygous genotypes remains consistently higher than expected. This frequency of homozygosity without any obvious loss of fitness may indicate both lineages encode receptors with equally important functions. Our data further demonstrate a higher amount of *KLRA* transcription in homozygotes, which is unrelated to the allele lineage. This trend is apparent in NCR^−^ and NCR1^+^ (with or without cytokine stimulation) and dilution culture datasets, suggesting a potential advantage of a homozygous genotype.

The lineages differ in their ligand binding domains, suggesting they may recognize different ligands, and the discrete patterns of transcription we observed would support this. Relatively higher levels of KLRA*02 are found under steady-state conditions (ex-vivo), with this trend reversed following cytokine stimulation. It is tempting to speculate the two cattle lineages contribute to opposing immune functions, which exist to balance one another. Rodent *KLRA* genes recognize MHC class I molecules, and although both cattle *KLRA* lineages are structurally related to their murine equivalents, sequence similarity is low. It has yet to be shown in cattle whether KLRA bind MHC class I or have evolved different ligand specificities.

The transcriptional regulation of *Ly-49/KLRA* genes in mice has been well characterized (14). Inhibitory KLRA are expressed in a monoallelic fashion (34), whereas biallelic expression of activating KLRA is apparent (35). Histone acetylation and DNA methylation of a downstream *Ly-49* promoter region Pro-2 underlies the regulation of *Ly-49a* monoallelic gene expression in mice (36). Our data show that cattle have biallelic expression at the lineage level, as heterozygotes always express both inhibitory KLRA*01 and KLRA*02 alleles that invariably contain intact ITIM sequence motifs. Although our quantitative PCR data are unable to determine whether both minor alleles in a homozygote are expressed, biallelic expression in heterozygotes and the higher level of transcription observed in homozygotes suggest monoallelic expression is unlikely in cattle.

Our wider analysis of *KLRA* in several divergent ruminant species revealed these lineages were present long before the speciation and subsequent domestication of cattle. This may be a rare example of *trans*-species polymorphism (37, 38). The apparent presence of only one *KLRA* lineage in the rest of the ruminant species we examined could be an artifact of low numbers of individuals or nonrandom deletion of a lineage. Regardless, these alleles have become fixed, and any intermediate alleles have either been lost through gradual selection or a recombination event. Although none of the other ruminant genomes we analyzed show more than one gene, there are species (human, horse, and pig) that only have KLRA*01-like genes, which may have emerged in a similar way. Further analysis of larger cohorts of the other ruminant species is needed to determine a more complete evolutionary history.

## Supplementary Material

Data Supplement
