# Supplementary material for: Two Lineages of KLRA with Contrasting Transcription Patterns Have Been Conserved at a Single Locus during Ruminant Speciation
Source: J Immunol. 2020 Mar 25;204(9):2455–63. doi: 10.4049/jimmunol.1801363 (PMC7167460; doi:10.4049/jimmunol.1801363)
Supplement: Data Supplement [file JI_1801363.zip › JI_1801363_Supplemental_Material_1.pdf]

Supplemental table 1. Comparison of log_2 normalised expression levels of KLRA1 and KLRA2 in *ex vivo* cells.

comparison	log_2 relative expression level†	adjusted *P*-value‡
KLRA1		
PBMC vs NCR1-	0.29	0.53
PBMC vs NCR1+	-2.44	<0.001
NCR1+ vs NCR1-	2.72	<0.001
homozygous vs heterozygous	1.89	0.01
KLRA2		
PBMC vs NCR1-	-1.18	0.02
PBMC vs NCR1+	-2.34	<0.001
NCR1+ vs NCR1-	1.16	0.02
homozygous vs heterozygous	1.84	0.06

† number of two-fold differences in expression of first group compared with second group

‡ Tukey's honest significant differences, which adjusts the *P*-value to allow for multiple testing

Supplemental table 2. Comparison of log_2 normalised expression levels of KLRA1 and KLRA2 in cytokine-stimulated cells.

comparison	log_2 relative expression level†	adjusted *P*-value‡
KLRA1		
NCR1+ vs IL-2	-0.86	0.17
NCR1+ vs IL-12/18	0.97	0.10
NCR1+ vs IL-15	-0.96	0.11
IL-2 vs IL-12/18	1.83	<0.001
IL2 vs IL-15	-0.09	0.99
IL-12/18 vs IL-15	-1.93	<0.001
homozygous vs heterozygous	2.18	<0.001
KLRA2		
NCR1+ vs IL-2	1.85	0.03
NCR1+ vs IL-12/18	0.98	0.45
NCR1+ vs IL-15	3.41	<0.001
IL-2 vs IL-12/18	-0.87	0.56
IL2 vs IL-15	1.56	0.14
IL-12/18 vs IL-15	2.42	0.005
homozygous vs heterozygous	3.02	<0.001

† number of two-fold differences in expression of first group compared with second group

‡ Tukey's honest significant differences, which adjusts the *P*-value to allow for multiple testing

Supplemental figure 1. Recombination has focussed at the telomeric end of the *KLRA* locus.

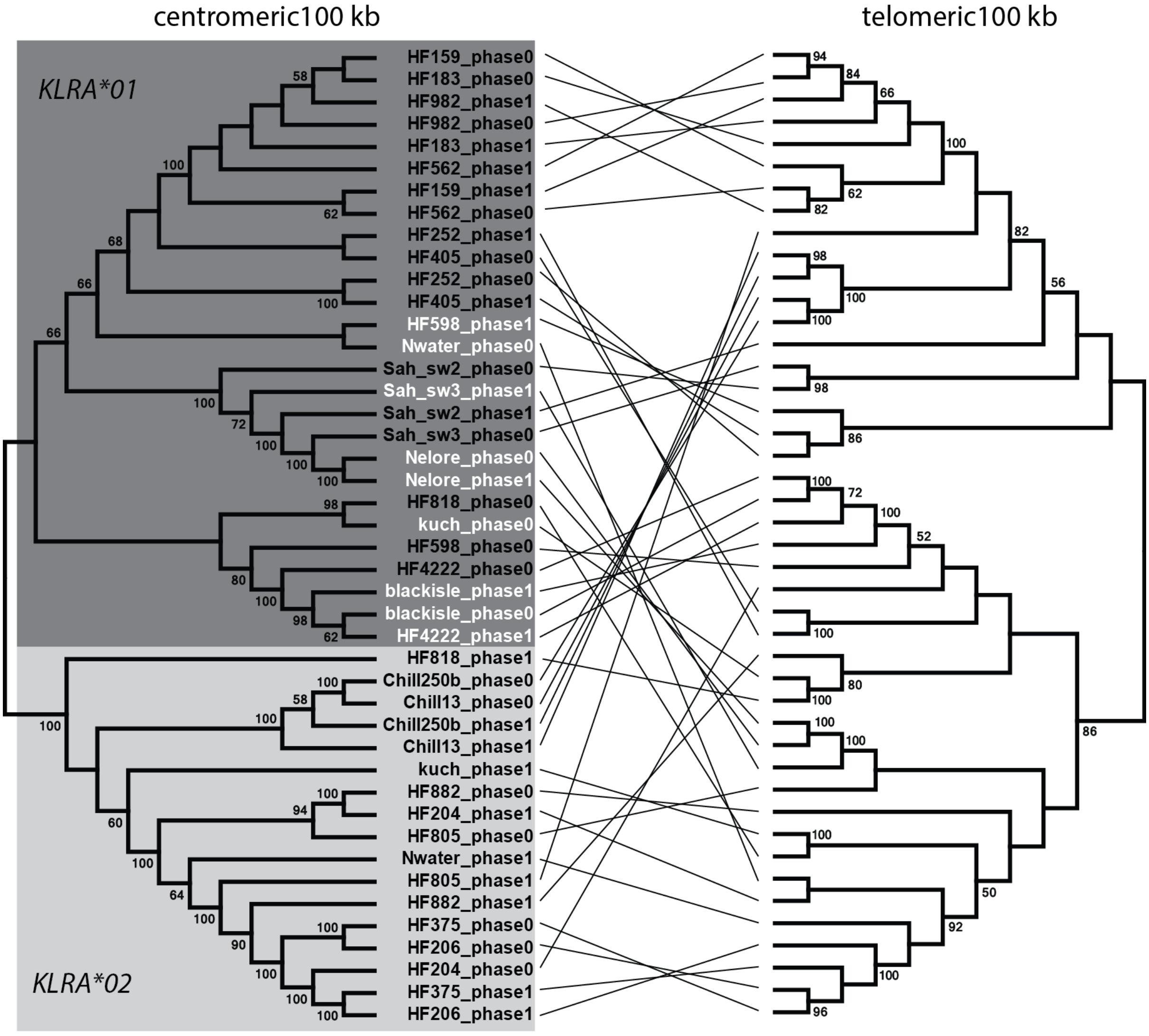

Phylogenetic analysis of phased sequences from probe enriched bovine genomes 100 kb either side of the *KLRA* locus. The tree was drawn in MEGA using the neighbour joining method with 100 bootstraps and rooted at the midpoint. Node support is shown when 50 % or above. Lines between each tree correspond to the position of sequences from the same allele from that animal. Recombinant alleles are highlighted by white text.

Supplemental figure 2. Cattle *KLRA*01* and *KLRA*02* transcripts can be expressed on the cell surface.

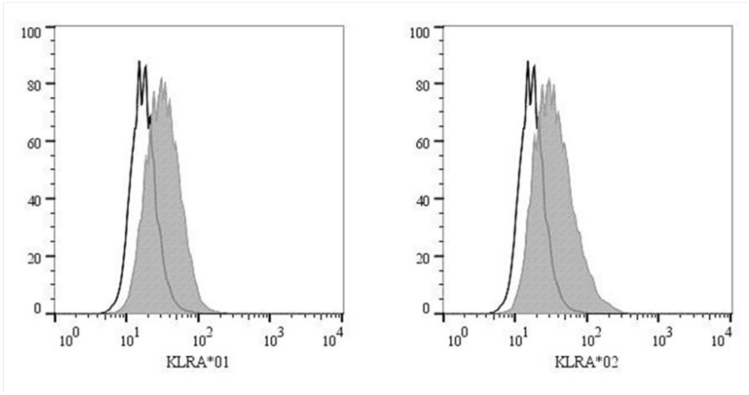

P815 cells transfected with *KLRA* constructs containing the V5 epitope were incubated with a mouse isotype control (open – black outline) or anti-V5 (shaded – grey outline) before washing and incubation with a PE-conjugated secondary antibody. P815 cells transfected with *KLRA* without the V5 epitope were used as a negative control. The expression of V5 was determined by flow cytometry, gating on the live cell population.
